# Successful treatment of dissecting cellulitis with certolizumab pegol in a pregnant patient

**DOI:** 10.1016/j.jdcr.2023.06.041

**Published:** 2023-07-08

**Authors:** Nouf Almuhanna, Rasha Alhamazani, Sarah Alkhezzi, Mahdi T. Alfataih, Alhanouf Bin Dakhil, Yasmin Saleh Alhamazani, Faris A. Alhomida

**Affiliations:** a Department of Dermatology, King Fahad Medical City, Prince Abdulaziz Ibn Musaid Ibn Jalawi St; b College of Medicine, University of Hail, Hail

**Keywords:** dissecting cellulitis, biologics, drugs, treatment, pregnancy, pregnant patient, hair, follicular disorders, immunomodulators, inflammation, follicular occlusion

## Introduction

Dissecting cellulitis of the scalp (DCS) is a rare, chronic, and often debilitating inflammatory disorder characterized by boggy, suppurative nodules of the scalp, often with scarring alopecia. Although its exact etiology is unknown, it is thought to be caused by follicular occlusion, with hyperkeratosis and accumulation of sebaceous and keratinous material that causes an intense inflammatory response, often with a superimposed bacterial infection. It is considered one of the follicular occlusion tetrads, along with hidradenitis suppurativa (HS), acne conglobata, and pilonidal cysts.^1^^,^^2^

Although the exact prevalence is unknown, DCS is classified as a rare disease by the National Institutes of Health. The literature suggests that it may be more common in young black males between the ages of 20 to 50 years. However, DCS cases in other ethnic groups, females, and younger patients have also been reported.^1^^,^^2^

DCS has traditionally been thought to be difficult to treat, with no agreed-upon standard treatment and no currently available Food and Drug Administration (FDA)-approved treatment.^1^^,^^2^

## Case report

**Fig 1:**
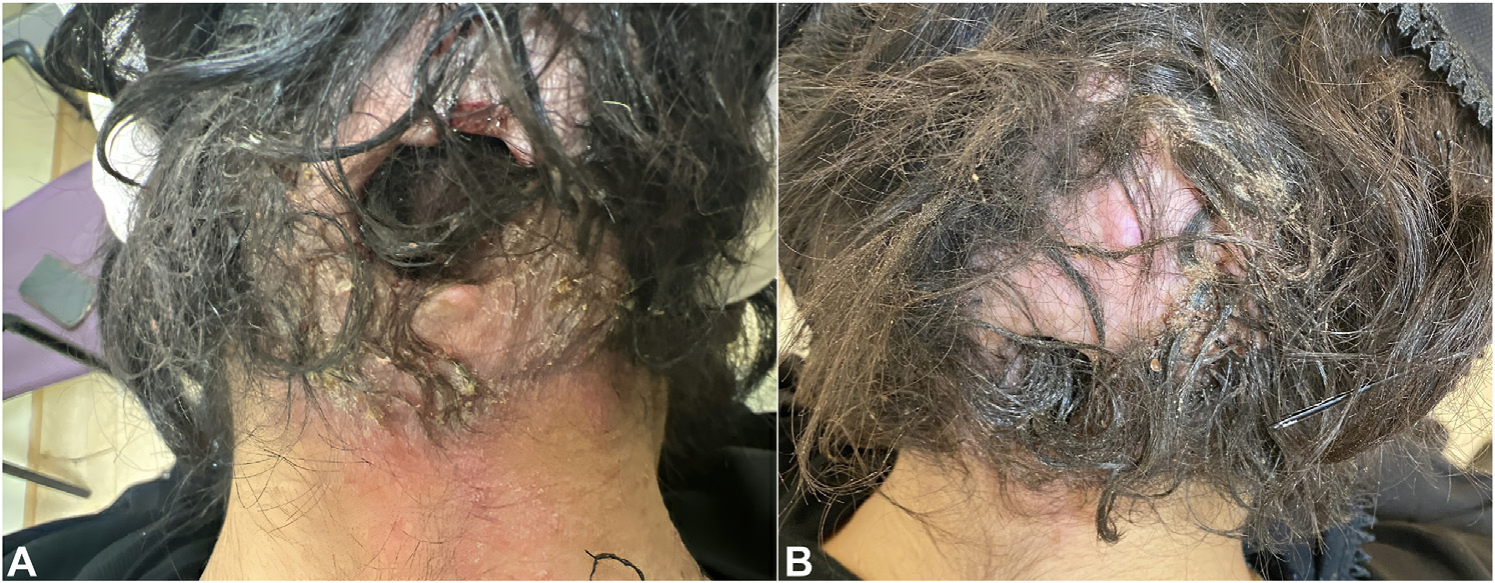
**A,** Before treatment with certolizumab pegol: An erythematous, boggy, hyperkeratotic, and atrophic plaque with overlying crust and surrounding alopecia of the occipital scalp and posterior neck. **B,** After treatment with certolizumab pegol: Reduction in the erythema, hyperkeratosis, and overlying crust is noted on the occipital scalp and posterior neck.

A 33-year-old woman, who was 12 weeks pregnant with a 5 year history of refractory dissecting cellulitis, was seen in our tertiary center dermatology. Examination was notable for diffuse, boggy, hyperkeratotic, and atrophic plaques with overlying crust, and scattered alopecic patches covered her scalp (Fig 1, *A*). She had no other relevant background medical history and was not on any medications besides prenatal vitamins. She had no other relevant family history and had no known drug allergies.

She had previously tried multiple conventional therapies, including topical and systemic antibiotics, systemic corticosteroids, and isotretinoin, without a significant improvement. Although serology testing for biologic treatment was pending, she was started on 500 mg of cephalexin 2 times a day for 10 days. The pretreatment laboratory analysis for the initiation of off-label certolizumab pegol revealed normal results for hepatitis B and C screening, HIV screening, QuantiFERON-TB Gold testing, complete blood count, comprehensive metabolic panel, lipid panel, liver, and renal function tests. The patient received off-label treatment with subcutaneous injections of certolizumab pegol with a loading dose of 400 mg at weeks 0, 2, and 4, followed by 200 mg every other week.

By 4-month follow-up, she reported a 70% improvement of her lesions, with less pruritus, tenderness, erythema, purulent discharge, crustation, and a reduction in the frequency of new lesions. By 8-month follow-up, she was now 4 weeks postpartum and continued to have a sustained treatment response and remained on certolizumab 200 mg every 2 weeks (Fig 1, *B*). No adverse reactions were noted.

## Discussion

DCS presents as a challenging entity, with no current FDA-approved treatment available. Conventional therapies that have been reported with some success include systemic antibiotics, isotretinoin, ablative lasers, and surgical excision.^1^^,^^2^ However, the treatment options for pregnant patients are more limited.

The tumor necrosis factor inhibition reduces inflammation in many immune-mediated diseases, including DCS. Adalimumab is a fully human, recombinant, antitumor necrosis factor-alpha (TNFα) immunoglobulin G1 monoclonal antibody that has been approved by the FDA for the treatment of adults and adolescents with HS.^3^ Given the similar pathogenesis of these follicular occlusive entities, it may present as a promising off-label treatment option for adult patients with DCS.^1^, ^3^, ^2^ However, adalimumab has been well-established as being able to cross the placenta, and it may be detected in the fetal circulation for up to 6 months postpartum.^4^ Thus, it may not be the ideal candidate for a pregnant patient.

A similar biologic agent, certolizumab pegol—a PEGylated, fragment crystallizable region (Fc) free, anti-TNFα, monoclonal antibody—has been approved by the FDA for the treatment of adult patients with Crohn disease, ankylosing spondylitis, rheumatoid arthritis, and psoriatic arthritis.^5^^,^^6^ Certolizumab, unlike other anti-TNFα biologics, lacks the Fc region of IgG and cannot bind to the neonatal Fc receptor (FcRn), which is needed for placental transfer. Thus, placental transfer is restricted. It has been safely used in pregnant patients with minimal, if any, placental transfer of the drug reported in the literature.^5^^,^^6^

To our knowledge, there are no other cases of DCS successfully treated with certolizumab reported in the literature to date, including pregnant patients. Our findings suggest that certolizumab may be a viable treatment option for pregnant patients with DCS. However, more research is warranted to further study the clinical effects of certolizumab for the treatment of DCS in the pregnant population.

## Conflicts of interest

None disclosed.
